# Supplementary material for: Priorities for Future Research About Screen Use and Adolescent Mental Health: A Participatory Prioritization Study
Source: Front Psychiatry. 2022 May 6;13:697346. doi: 10.3389/fpsyt.2022.697346 (PMC9120839; doi:10.3389/fpsyt.2022.697346)
Supplement: Supplementary file 2 [file Presentation_1.pdf]

Screen use and young people's mental health

Project findings

The process

1. Consulting researchers in the field.
2. Discussion groups with young people, parents and teachers.
3. Public consultation: ranking important questions.

Final list of important questions

Which questions were voted top?

- What is the relationship between screen use and mental health and wellbeing for young people from vulnerable groups (e.g., mental or physical health conditions, disability, learning difficulties)?
- What is the impact of screen use on brain development?
- What impact does exposure to adult content (e.g., violent, sexual) have on young people's mental health and relationships to others?
- What is the relationship between screen use, sleep and mental wellbeing in young people?

Overall, people prioritised:

- Exposure to adult content online
- Wellbeing of vulnerable populations
- Impact of screen use on development
- The effect of screen use on sleep

Young people prioritised:

- Developing mental health problems through social media
- Impact of bullying online
- Companies exploiting adolescent’s vulnerabilities

Public consultation survey

229 young people and 128 adults completed the survey.

87%	6%	5%
England	Scotland	Wales

Ethnicity

78%	White British/European
10%	Asian/Asian British
6%	Mixed/Multi Ethnic group
5%	Black/African/Black British/Caribbean
1%	Other

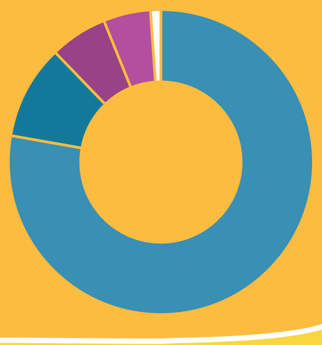

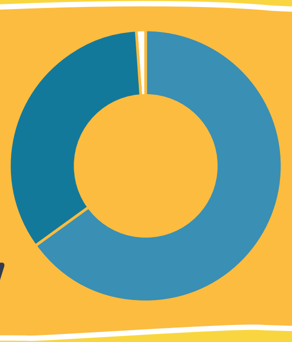

Gender

65%	Female
34%	Male
1%	Other

The McPin Foundation Young People's Advisory Group were involved in:

- Analysis and interpretation
- Recruitment strategy
- Data collection materials
- Facilitating discussion groups
- Attending the steering group meetings
- Making key decisions about the project throughout

Impacts of lockdown

Participants' views on screen use and young people's mental health became more favourable during lockdown, potentially due to greater reliance on screens for education, communication, and entertainment and other significant events including the Black Lives Matter movement.

Research topics of interest arising during this time included the effects on adolescent mental health of exposure to constant news updates and online racial bias, and how young people take part in activism online.

Next steps

This work points to the great need for more evidence in this field, including potential risks and benefits at stake of social media use. Findings should inform calls for research and funding allocation in order to develop evidence-based mental health policy and guidelines.

Mental Health Policy Research Unit mhpru
McPin Foundation Transforming mental health research
Young People's Network
Centre for Mental Health
